# The New Physiology in Surgical and General Practice

**Published:** 1913-03

**Authors:** 


**The New Physiology in Surgical and General Practice.** By A. RENDLE SHORT, M.D., B.S., B.Sc. Lond., F.R.C.S. Eng. Second Edition. Pp. xi, 244. Bristol: John Wright and Sons Ltd. 1912. 5s.

Dr. Short has opened out quite a new line in this little work, and the profession have not been slow in appreciating its utility. Hence the exhaustion of the first edition in a few months, and the necessity for the present volume. Research speeds apace in the subjects dealt with and although so short a time has elapsed, a number of additions have been made. A chapter on the growth of bone has been added embodying Macewen's recent work, showing that the periosteum takes no share in the regeneration of bone. Henderson's views on the relation of a diminished quantity of $CO_2$ in the blood to shock, certain risks attaching to the administration of saline infusions, and the movements of digestion are further additions to note. New paragraphs have been added dealing with gastric secretion in man, the pituitary gland and the use of pituitary extract, sensory localisation in the brain, the innervation of the pleura and the referred pain of pleuritis and fibrositis. It is impossible to speak in too high terms of this book. It is a most lucid exposition of work much of which is none too easy to grasp, and which was unknown to those who finished their medical curriculum twenty years ago.

